# German language adaptation of the Cluster Headache Quality of Life Scale (CH-QoL)

**DOI:** 10.1186/s12883-024-03923-6

**Published:** 2024-11-07

**Authors:** Hannah Kuhn, Tara Petzke, Marie-Christin Schreiber, Charly Gaul, Michael Witthöft, Timo Klan

**Affiliations:** 1 Department of Psychology, Johannes Gutenberg-University of Mainz, Wallstr. 3, 55099 Mainz, Germany; 2 Headache Center Frankfurt, Frankfurt, Germany

**Keywords:** Cluster headache, Quality of life, Questionnaire, Trigeminal autonomic cephalalgia, Patient-reported outcome measure

## Abstract

**Background:**

Cluster headache (CH) can lead to high disability and reduced quality of life (QoL). QoL should be assessed as an important outcome both in research and in clinical care. The 28-item Cluster Headache Quality of Life Scale (CH-QoL) is a valid self-report questionnaire to assess disease-specific QoL. A German version is lacking. This study aims to develop a German-language version and to determine its psychometric properties.

**Methods:**

The CH-QoL was translated into German by two headache experts and blindly back-translated by two professional translators. Additionally, the CH-QoL was tested for comprehensibility by nine persons with CH. In this multi-stage process, linguistic discrepancies were repeatedly discussed in an expert panel and appropriate modifications were conducted to optimize the translation. A cross-sectional online survey, comprising the CH-QoL and further self-report questionnaires such as the Cluster Headache Scales (CHS), yielded a sample of *N* = 106 persons with CH (53.8% female, *M* = 45.5 [*SD* = 11.8] years, 48.1% episodic CH, 51.9% chronic CH, 79.2% currently having recurring CH attacks).

**Results:**

Exploratory factor analysis revealed two clearly interpretable factors (“restriction of activities of daily living”, and “impact on mood and interpersonal relationships”), which is in discrepancy to the four factors of the original English version. The model fit was good, with χ^2^(323) = 590.74, *p* < .001, *RMSEA* = 0.088, *SRMR* = 0.053, *TLI* = 0.857. Reliability was very good (McDonald’s omega ω = 0.97, Subscale/Factor 1: ω = 0.96, Subscale/Factor 2: ω = 0.92). Correlational analyses (correlations with related questionnaires as well as with clinical parameters) confirmed convergent validity.

**Conclusions:**

Since the German version of the CH-QoL has very good psychometric properties, it is suitable for the assessment of disease-specific QoL in people with CH in German-speaking countries.

**Trial registration:**

This study is registered with the German Clinical Trials Register (DRKS-ID: DRKS00028475, registration date 03 March 2022).

**Supplementary Information:**

The online version contains supplementary material available at 10.1186/s12883-024-03923-6.

## Background

Cluster headache (CH) is a primary headache disorder, characterized by recurring attacks of unilateral, severe headache, associated with autonomic symptoms and restlessness or agitation [1]. The prevalence of CH is between 0.1 and 0.2% [2]. CH can lead to high disability and reduced quality of life (QoL), especially in persons with chronic CH [3]. QoL is also impaired during attack-free periods or outside of cluster episodes [4, 5]. The risk of developing a mental disorder is significantly higher compared to people without CH [6, 7]. For example, the probability of developing a depressive disorder is up to three times higher [8], and the extent of suicidal tendencies is also increased [4, 9]. Disability is also higher compared with other headache disorders [3]. Thus, health-related QoL and disability are important clinical outcomes beyond the recording of pain symptoms. Since QoL and disability questionnaires make it possible to assess the impact of a disease on well-being they should be used in treatment studies for persons with chronic pain [10]. One criticism of generic questionnaires for assessing QoL (such as the SF-36 Health Survey Questionnaire) is that specific characteristics of the disease (in the case of CH, e.g., changes in appearance during the CH attack due to swelling of the eyes) are not adequately addressed. It therefore makes sense to develop specific measurement instruments that take these specific characteristics into account. There are currently three different self-report questionnaires for the disease-specific assessment of the impact of CH, (i) the Cluster Headache Scales (CHS) [11], (ii) the Cluster Headache Impact Questionnaire (CHIQ) [12], and (iii) the Cluster Headache Quality of Life Scale (CH-QoL or CHQ) [13, 14], each having a different focus. With eight subscales (“medical care”, “medication side effects”, “fear of attacks”, “disability”, “(auto)aggression”, “coping”, “physical activity”, and “financial burden”), the CHS covers a broad spectrum of psychosocial burden in persons with CH. The CHIQ focuses on CH-related impairment with only one scale. The CH-QoL captures various aspects of QoL with four subscales (“restriction of activities of daily living”, “impact on mood and interpersonal relationships”, “pain and anxiety”, and “lack of vitality”). All three questionnaires demonstrated good psychometric properties in cross-sectional validation studies [11–13]. Sensitivity to change was also demonstrated for the CH-QoL [14]. Since the CH-QoL and the CHS are in part complimentary, both scales are recommended as patient-reported outcome measures (PROMs) in the current guidelines of the International Headache Society for controlled clinical trials in CH [15].

With a prevalence of 0.12% [16], CH is also relevant to healthcare in Germany. However, a German-language version of the CH-QoL is not yet available. The present study aims to develop an adequate German-language version and to determine both the factor structure and the psychometric properties.

## Methods

### Study design, participants, and procedure

The study was conducted as a cross-sectional online survey via SoSci Survey (https://www.soscisurvey.de) [17] by the Department of Psychology of the Johannes Gutenberg-University of Mainz, Germany. The Ethics Committee of the Department of Psychology (Johannes Gutenberg University of Mainz) approved the research protocol (2022-JGU-psychEK-005). The study was prospectively registered with the German Clinical Trials Register (https://drks.de/search/de, DRKS-ID: DRKS00028475, registration date: 03 March 2022).

Inclusion criteria were (i) age of at least 18 years, (ii) diagnosis of CH (made by a physician), and (iii) duration of CH of at least 12 months (i.e., the disease must have existed for at least 12 months). Participants with a conspicuously fast processing time (relative speed index [RSI] > 2) [18] or with an incorrect response to an instructed response item (IRI) [19] were excluded post-hoc from the analysis. The RSI is computed by SoSci Survey. In doing so, the median processing time across all participants is calculated for each page of the survey. For each participant and each page, this median is then divided by the individual processing time on that page. To determine the RSI of a participant, the average of these speed factors across all pages is calculated. It is recommended to remove all participants with an RSI > 2 from the data set, as such a high RSI suggests that not all questions were answered carefully [18]. IRIs are used to check the attention of participants while completing a survey [19]. In our study, we placed the IRI after the 28 items of the CH-QoL, instructing the participants to tick the response category “occasionally”. Any participant who answered the IRI incorrectly (i.e., ticked any other response instead of “occasionally”) was excluded.

Participants were recruited via different websites (i.e., German Association of Cluster Headache Self-help Groups, German Migraine and Headache Society), three headache clinics or centers (Migraine and Headache Clinic Königstein, Bavarian Headache Center München, Headache Center Frankfurt), and social media (i.e., Facebook self-help group for CH). To determine the sample size, the rule of thumb was applied to aim for at least 10 participants per item if an exploratory factor analysis (EFA) is to be carried out [20]. Since the CH-QoL comprises 28 items, a sample size of *N* = 280 was aimed for.

At the beginning of the survey, the participants had to tick a checkbox to confirm that they met the inclusion criteria and that they consent to take part in the study. After that, socio-demographic and disease data (e.g., disease duration, treatment) were queried. The participants were then presented with the CH-QoL (German version). To be able to assess the validity of the CH-QoL, further self-report questionnaires (cf., measures) followed. At the end of the survey, the participants declared whether they had completed the survey conscientiously as well as whether their data may be stored and used for scientific purposes.

### Development of a German-language version

**Fig. 1:**
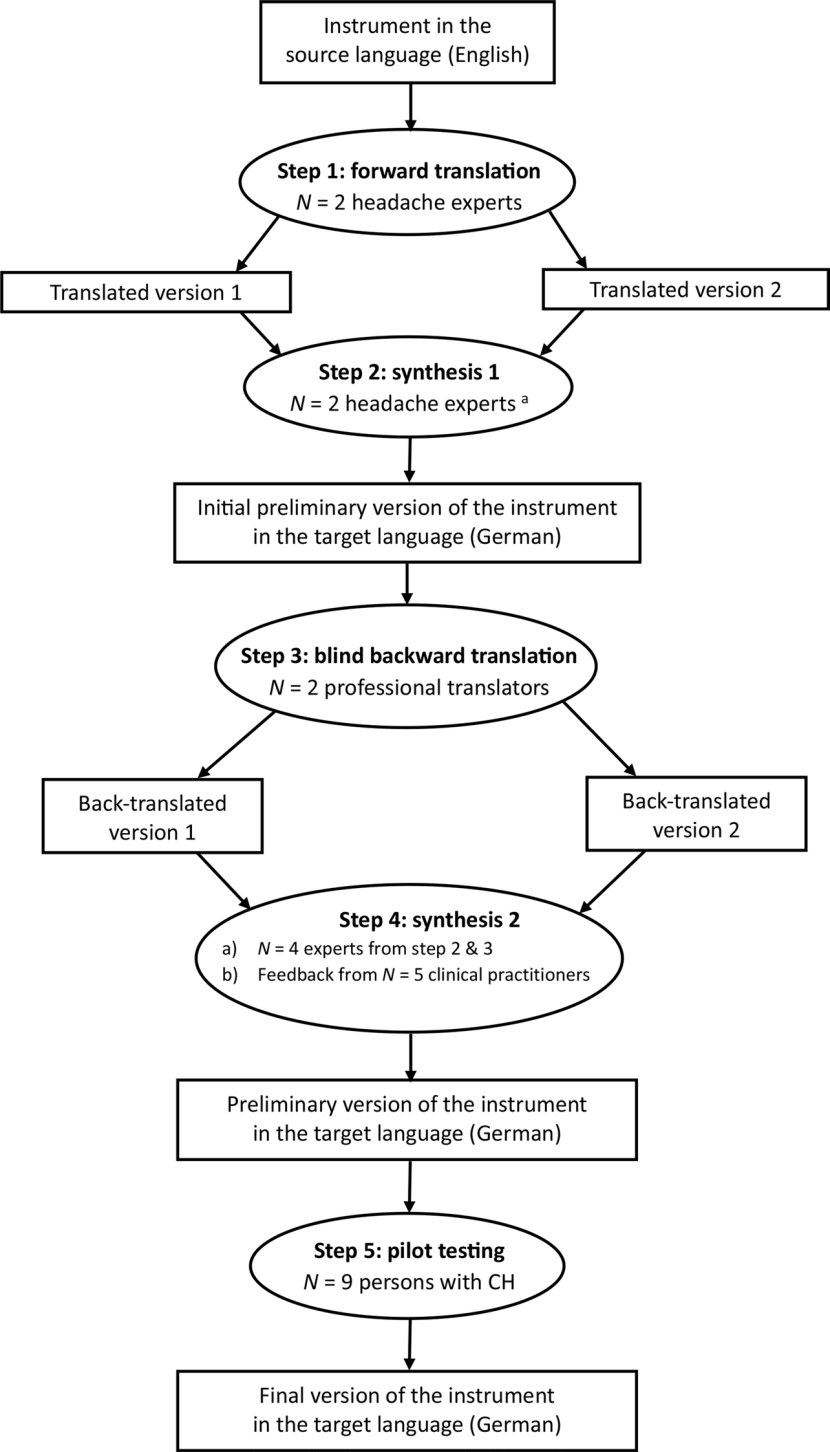
Translation process. ^a^ same persons as in step 1. CH Cluster headache

The German translation of the CH-QoL was based on a guideline for the translation, adaptation, and validation of research instruments [20]. First, two native German speakers, each with headache expertise, translated the CH-QoL into German independently of each other (forward translation, Fig. 1). Second, the two translations were compared, modified, and combined into one German translation (synthesis 1). Third, this German version was blindly translated back into English independently by two native English speakers, both bilingual professional translators with experience in the translation of headache-related text. In a fourth step (synthesis 2), both backward translations were compared with each other as well as with the CH-QoL. Four experts (the persons from step 2 and 3) developed a preliminary German version of the CH-QoL, involving the feedback from five clinical practitioners (*n* = 2 physicians, *n* = 3 psychologists, each with expertise in the treatment of CH). The preliminary German version of the CH-QoL was then tested for comprehensibility and relevance in a sample of nine CH patients (step 5, pilot testing), which resulted in the final German version of the CH-QoL (Supplementary Fig. S1). As with the original English version (Supplementary Fig. S2), the German version has 28 items, which measure how often CH has affected various aspects of life during the last month. Each item is answered on a 5-point scale (0 = never, 1 = occasionally, 2 = sometimes, 3 = often, 4 = always). The total score of the questionnaire is the sum of all 28 items, with higher values mirroring lower QoL. The CH-QoL ends with an additional question in which overall life satisfaction is assessed on a visual analog scale (VAS, from 0 to 100, 0 = extremely dissatisfied, 100 = extremely satisfied).

### Measures

To assess the convergent validity of the CH-QoL we used the Short Form 36 Health Survey Questionnaire (SF-36) [21, 22], the Migraine-Specific Quality of Life Questionnaire (MSQ v2.1) [23, 24], the EuroQoL 5 Dimensions Questionnaire (EQ-5D-3L) [25, 26], the Cluster Headache Scales (CHS) [11], and the Cluster Headache Impact Questionnaire (CHIQ) [12] in the online survey (each the German version). Divergent validity was examined using the subscale “Openness” of the NEO-Five-Factor-Inventory (30-Item-Short-Version, NEO-FFI-30) [27].

#### Short Form 36 Health Survey Questionnaire (SF-36)

The SF-36 assesses general health-related QoL, referred to the last month on the basis of 36 items [21, 22]. There are 11 different response scales, therefore the item values are initially recoded to values from 0 to 100 with higher values reflecting higher QoL. The questionnaire consists of eight subscales: (1) Physical functioning (PF), (2) Role physical (RP), (3) Bodily pain (BP), (4) General health (GH), (5) Vitality (VT), (6) Social functioning (SF), (7) Role emotional (RE), (8) Mental health (MH). In addition, the authors of the SF-36 use two summary measures to aggregate the eight subscales of the SF-36: the Physical Health sum scale (PCS), consisting of subscales 1 to 4, and the Mental Health sum scale (MCS), consisting of subscales 5 to 8 [22]. The SF-36 has good reliability and validity [21, 22].

#### Migraine-Specific Quality of Life Questionnaire Version 2.1 (MSQ v2.1)

The MSQ v2.1. consists of 14 items on a 6-point scale and measures migraine-specific QoL, referred to the last month [23]. All item values are transformed into values from 0 to 100, with higher values mirroring higher QoL. To adapt the MSQ v2.1 to the target group of the survey, the term *migraine* was replaced by *cluster headache* in this questionnaire. In doing so, we followed the procedure of the study by Ertsey and colleagues, who substituted the term “migraine” with “headache attack” in the MSQ instruction to adequately assess QoL in three subgroups (persons with migraine, persons with CH, and a control group without a headache disorder) [28]. The MSQ v2.1 consists of 3 subscales: (1) Role function restrictive (RFR), (2) Role function preventive (RFP), and (3) Emotional functioning (EF). It is considered reliable and valid [23].

#### EuroQoL 5 Dimensions Questionnaire, 3-Level-Version (EQ-5D-3L)

The EQ-5D-3L assesses the general health state, referring to the current day. It consists of 5 items that are answered on a 3-point scale [25]. Each item measures a different health dimension (Mobility, Self-care, Usual Activities, Pain/Discomfort, and Anxiety/Depression), with higher values reflecting higher impairment of health. In addition, the general health on the day of the survey is assessed on a VAS from 0 (The worst health you can imagine) to 100 (The best health you can imagine). Reliability and validity were confirmed for various diseases [29, 30].

#### Cluster Headache Scales (CHS)

The CHS was designed to measure psychosocial burden in persons with CH, with higher values reflecting higher burden or lower coping skills [11]. This 36-item questionnaire is responded to on a 5-point scale, each referring to the current life situation. It has 8 subscales: [deficient] Medical care, Medication side effects, Fear of attacks, Disability, (Auto)Aggression, [deficient] Coping, [deficient] Physical activity, and Financial burden. The questionnaire has good reliability and validity [11].

#### Cluster Headache Impact Questionnaire (CHIQ)

The CHIQ assesses CH-specific disability, with higher values reflecting higher disability [12]. It comprises 8 items with a 6-point answering format (“never” to “always”), each referring to the last week. The questionnaire has good reliability and validity [12].

#### Openness

As a measure of divergent validity, we used the subscale “Openness” of the NEO-Five-Factor-Inventory (30-Item-Short-Version, NEO-FFI-30) [27]. This subscale comprises 6 items with a 5-point answering scale, it aims to assess the disposition to be open towards new experiences. The psychometric properties of the NEO-FFI-30 have been confirmed, with a tendency to be better compared with the long version [27].

### Statistical analyses

All statistical analyses were conducted using R version 4.3.0 and R studio version 2023.03.1 + 446 [31, 32]. Only the data of the participants were used who had given their consent at the beginning as well as at the end of the survey.

To test the suitability of the data for EFA, the Kaiser-Meyer-Olkin criterion as well as Bartlett’s test of sphericity were applied. The EFA was conducted with the minimum residual (MINRES) factoring method and direct oblimin rotation. Kaiser-Guttman criterion, the scree plot, and Horn’s parallel analysis were used to determine the number of factors. The model fit was assessed with the chi-square test, the root mean square error of approximation (*RMSEA*), the standardized root mean square residual (*SRMR*), and the Tucker-Lewis Index (*TLI*) [33].

To estimate the reliability of the CH-QoL, McDonald’s Omega ω was calculated. Convergent and divergent validity were tested with Pearson correlations between the CH-QoL (total scores as well as subscale scores) and the other questionnaires (cf. section on Measures) except for the EQ-5D-3L. Due to the ordinal scales of the EQ-5D-3L, Spearman correlations were calculated. A significance level of α = 0.05 was set for all correlations (two-tailed). To further examine the construct validity of the CH-QoL, a Pearson correlation (two-tailed, significance level of α = 0.05) between the CH-QoL total scores and the number of CH attacks during the last month was calculated.

Additionally, group comparisons were conducted regarding current disease activity (currently no CH attacks vs. recurrent attacks) and the subtype of CH (episodic vs. chronic CH). Since the prerequisite of variance homogeneity was not fulfilled (disease activity: Levene test: *F*(1,104) = 15.356, *p* < .001; subtype of CH: Levene test: *F*(1,104) = 4.839, *p* = .030), we conducted Welch *t*-tests instead of regular *t*-tests to compare each the mean CH-QoL total scores (two-tailed, significance level of α = 0.05).

## Results

### Participants

**Fig. 2:**
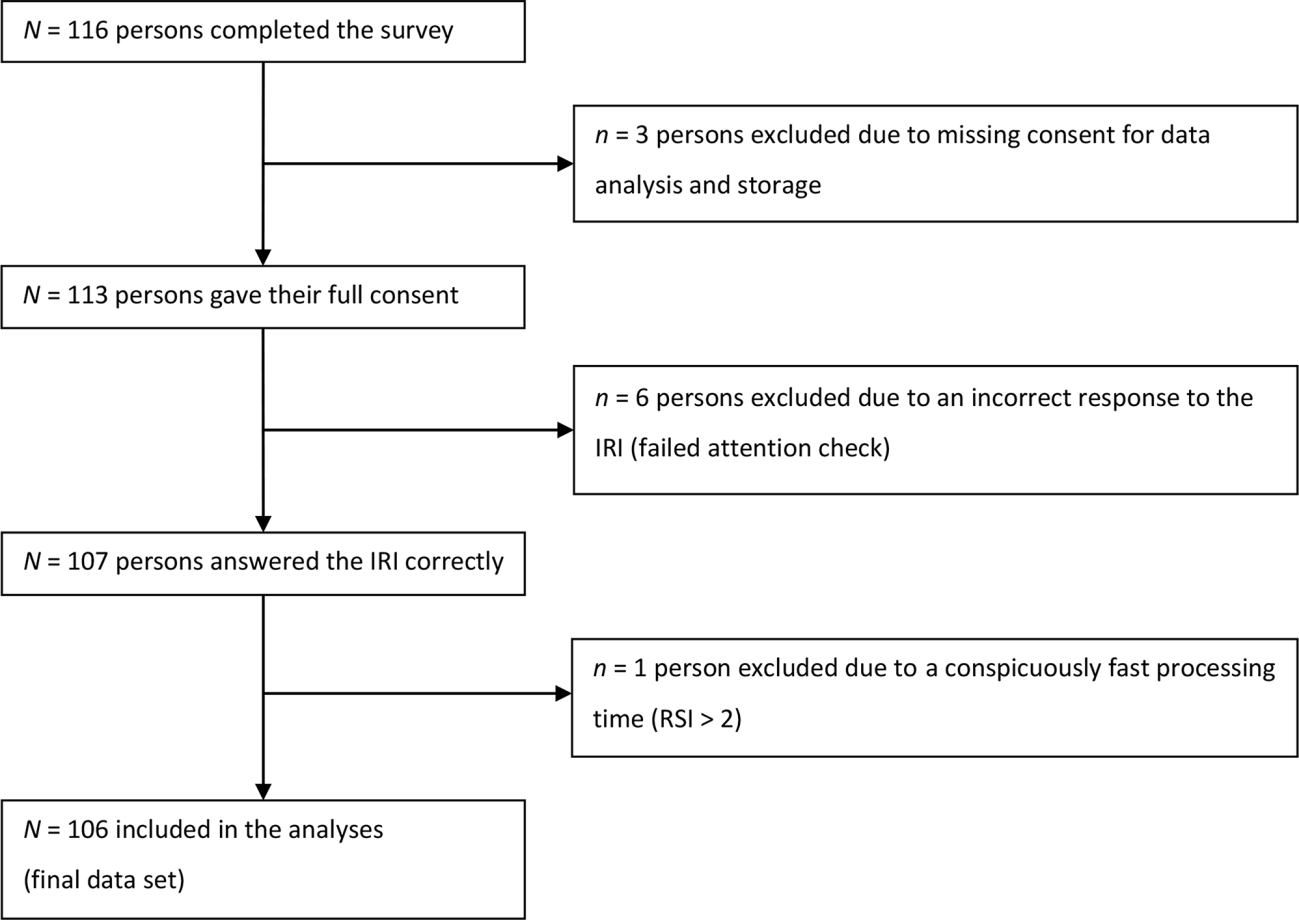
Flowchart of attrition. IRI instructed response item, RSI relative speed index

Participants were recruited from March 03, 2023, to May 05, 2023. We received complete data from *N* = 116 persons (Fig. 2). Of these, *n* = 3 did not consent to their data being analyzed. Furthermore, *n* = 6 failed the IRI attention check item, and *n* = 1 other person was excluded because they were too fast (RSI > 2). Subsequently, our final data set consisted of *N* = 106 persons (Table 1).

**Table 1:** Sample characteristics

	*N* = 106
Age (years)	45.5 (11.8)
Female	57 (53.8%)
Education level (tertiary entrance degree or higher)	54 (50.9%)
Currently in a partnership	78 (73.6%)
Subtype of CH	
Episodic	51 (48.1%)
Chronic	55 (51.9%)
Disease activity	
Currently no attacks	22 (20.8%)
Currently recurring attacks	84 (79.2%)
Disease duration (years)^a^	9.8 (7.8)
Acute attack medication^b^	
Oxygen	73 (68.9%)
Sumatriptan	67 (63.2%)
Zolmitriptan	42 (39.6%)
Lidocaine/Procaine	4 (3.8%)
No acute attack medication	1 (0.9%)
Medication prophylaxis	67 (63.2%)
Smoker	65 (61.3%)
Trigger of CH attacks^b^	
Alcohol	57 (53.8%)
Emotional distress	51 (48.1%)
Weather/change of weather	50 (47.2%)
Flickering light	39 (36.8%)
Lack of sleep	34 (32.1%)
Heat	28 (26.4%)
Histamine	27 (25.5%)
Nicotine	4 (3.8%)
Caffeine	4 (3.8%)
Menstruation	4 (3.8%)
Else	22 (20.8%)

*Note* Data are *n* (%) or *M* (*SD*)

^a^Disease duration since first CH diagnosis

^b^Multiple answers possible

### Factorial structure and model fit

**Fig. 3:**
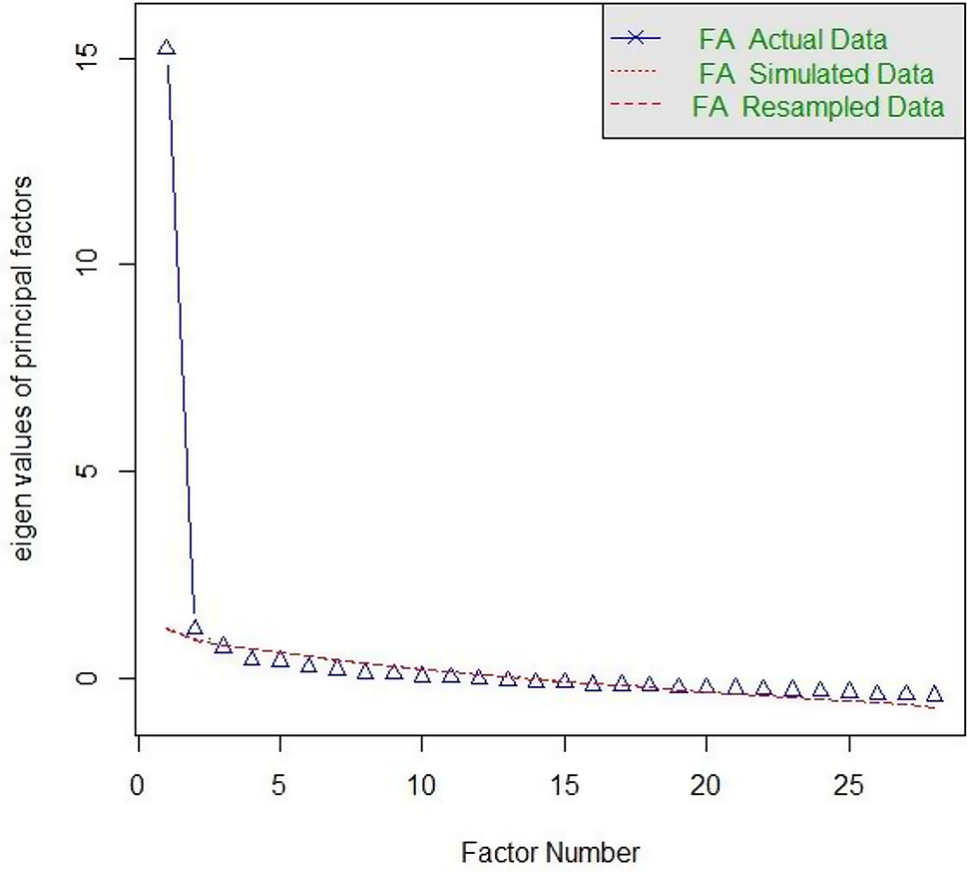
Scree plot with observed eigenvalues (EFA) and parallel analysis

The Kaiser-Meyer-Olkin criterion indicated good suitability for factor analysis at MSA = 0.94, and Bartlett’s test of sphericity was significant (χ^2^(378) = 2612.33, *p* < .001). In the scree plot (Fig. 3) and parallel analysis, all criteria pointed towards two factors. For the Kaiser-Guttman criterion, two factors had eigenvalues above 1; the “elbow” of the scree plot was at two factors (Fig. 3); and the parallel analysis suggested the extraction of two factors.

Therefore, we conducted a two-factor EFA with direct oblimin rotation (MINRES method). The model fit was good, with χ^2^(323) = 590.74, *p* < .001, *RMSEA* = 0.088, *SRMR* = 0.053, *TLI* = 0.857. The first factor had an eigenvalue of λ_1_ = 10.72, and the second factor had an eigenvalue of λ_2_ = 5.83, which means a total of R² = 59.11% of the variance was explained. The factors correlated highly at *r* = 0.76, indicating that oblique rotation would be more suitable than orthogonal rotation. For the rotated factor loadings, see Table 2. While most items could be assigned to one of both factors, Items 20 and 21 showed only mediocre loadings on both factors. Item 20 was assigned to subscale 1 and Item 21 to subscale 2, as the loadings on these factors were each minimally higher.

**Table 2:** MINRES-rotated factor solution for the 28 CH-QoL items

Item	Factor 1	Factor 2
Due to cluster headache, during the last month how often have you:		
1. Avoided leaving the house	0.90	
2. Avoided making plans due to unpredictability of cluster headache (e.g. holidays)	0.64	
3. Felt unable to complete duties at work	0.72	
4. Had difficulty in getting involved in leisure activities (e.g. go to the movies, theatre)	0.89	
5. Avoided crowded and noisy places (e.g. restaurants, public transport)	0.65	
6. Felt that the severity of cluster headache affected your daily activities	1.00	
7. Been less involved in family affairs (e.g. interaction with children, planning vacations)	0.76	
8. Been unable to socialize/spend time with friends and family	0.86	
9. Been unable to achieve your daily goals and carry out routines and chores	0.88	
10. Felt less respected by others		0.78
11. Had problems with close personal relationship		0.82
12. Felt you were a burden on family and friends		0.63
13. Felt self-conscious and uncomfortable about your appearance after a cluster headache attack (e.g. swelling/redness of eyes and facial sweating)		0.48
14. Felt that others are dismissive of your cluster headaches		0.56
15. Felt aggressive		0.72
16. Felt bad about yourself, lost self-confidence or felt worthless		0.83
17. Felt like harming yourself or suicidal		0.48
18. Been irritable, impatient or less tolerant		0.50
19. Been forgetful (e.g. missed appointments)	0.49	
20. Been unable to take care of your appearance (e.g. take a bath or shower, put make-up on, change clothes)	0.42	0.34
21. Felt isolated, lonely or vulnerable	0.37	0.44
22. Found your pain is unbearable if untreated	0.68	
23. Dreaded that the headache would not go away.	0.38	
24. Felt lacking in energy and constantly tired	0.83	
25. Felt sleepy, worn out or less able to concentrate due to nocturnal attacks of cluster headache	0.67	
26. Had problems concentrating (e.g. reading newspaper, watching TV)	0.85	
27. Been unable to think clearly	0.70	
28. Felt tense or anxious		0.63

*Note* Only loadings > ± 0.30 are displayed

### Reliability and validity

Regarding the internal consistency of the total questionnaire, McDonald’s omega was very good at ω = 0.97. Both subscales individually also showed high internal consistency (ω_Sub1_ = 0.96 and ω_Sub2_ = 0.92).

Table 3 shows the correlations between the CH-QoL subscales and total scores with the SF-36, MSQ, EQ-5D-3L, CHS, CHIQ, and the VAS at the end of the CH-QoL. Most correlations were high, statistically significant, and went in the hypothesized direction. As expected, the impact of having CH attacks (assessed by the CHIQ), and psychosocial burden of CH (assessed by the CHS) correlated positively with the CH-QoL, while health-related QoL (assessed by the SF-36), headache-specific QoL (assessed by the modified MSQ v2.1), and the general health state (assessed by the EQ-5D-3L) were correlated negatively.

**Table 3:** Correlation table including convergent and divergent validity measures

	CH-QoL Total *r* (*p*-value)	CH-QoL Subscale 1 *r* (*p*-value)	CH-QoL Subscale 2 *r* (*p*-value)
Convergent Validity			
SF-36			
* Physical Health sum scale (PCS)*	–0.78 (< 0.001)		
* Mental Health sum scale (MCS)*	–0.78 (< 0.001)		
* Physical functioning (PF)*		–0.49 (< 0.001)	–0.50 (< 0.001)
* Role physical (RP)*		–0.62 (< 0.001)	–0.57 (< 0.001)
* Bodily pain (BP)*		–0.80 (< 0.001)	–0.62 (< 0.001)
* General health (GH)*		–0.37 (< 0.001)	–0.36 (< 0.001)
* Vitality (VT)*		–0.70 (< 0.001)	–0.59 (< 0.001)
* Social functioning (SF)*		–0.78 (< 0.001)	–0.70 (< 0.001)
* Role emotional (RE)*		–0.50 (< 0.001)	–0.59 (< 0.001)
* Mental health (MH)*		–0.65 (< 0.001)	–0.68 (< 0.001)
MSQ v2.1			
MSQ total score	–0.87 (< 0.001)		
* Role function restrictive* (*RFR*)		–0.88 (< 0.001)	–0.72 (< 0.001)
* Role function preventive* (*RFP*)		–0.81 (< 0.001)	–0.66 (< 0.001)
* Emotional functioning* (*EF*)		–0.71 (< 0.001)	–0.76 (< 0.001)
EQ-5D-3 L			
* Mobility*		0.30 (0.002)^a^	0.39 (< 0.001)^a^
* Self-care*		0.13 (0.174)^a^	0.10 (0.298)^a^
* Usual activities*		0.36 (< 0.001)^a^	0.33 (< 0.001)^a^
* Pain/discomfort*		0.33 (< 0.001)^a^	0.29 (0.003)^a^
* Anxiety/depression*		0.48 (< 0.001)^a^	0.46 (< 0.001)^a^
* General health*	–0.54 (< 0.001)		
CHS			
CHS total score	0.68 (< 0.001)		
* [deficient]Medical care*		0.27 (0.006)	0.21 (0.030)
* Medication side effects*		0.19 (0.055)	0.27 (0.006)
* Fear of attacks*		0.32 (< 0.001)	0.45 (< 0.001)
* Disability*		0.73 (< 0.001)	0.66 (< 0.001)
* (Auto)Aggression*		0.19 (0.045)	0.43 (< 0.001)
* [deficient] Coping*		0.23 (0.018)	0.18 (0.063)
* [deficient] Physical activity*		0.24 (0.013)	0.28 (0.003)
* Financial burden*		0.37 (< 0.001)	0.47 (< 0.001)
CHIQ (total score)	0.85 (< 0.001)		
CH-QoL Overall life satisfaction (visual analog scale from 0 to 100)	–0.68 (< 0.001)		
Divergent Validity			
* Openness* (NEO-FFI-30)	0.16 (0.096)	0.17 (0.078)	0.13 (0.192)

*Note* All correlations are Pearson correlations unless otherwise stated. To adapt the MSQ v2.1 to the target group of the study, the term “migraine” was replaced by “cluster headache” in the instruction

CH-QoL Cluster Headache Quality of Life Scale, SF-36 Short Form 36 Health Survey Questionnaire, MSQ Migraine-Specific Quality of Life Questionnaire, EQ-5D-3L EuroQoL 5 Dimensions Questionnaire, CHS Cluster Headache Scales, CHIQ Cluster Headache Impact Questionnaire, NEO-FFI-30 NEO-Five-Factor-Inventory (30-Item-Short-Version)

^a^ Spearman correlations

Further, there were no significant correlations between the CH-QoL and the subscale “Openness” of the NEO-FFI-30, our measure of divergent validity (*r*_tot_ = 0.16, *p* = .096, *r*_Sub1_ = 0.17, *p* = .078, and *r*_Sub2_ = 0.13, *p* = .192).

### Subgroup analyses

We compared people with recurring CH attacks (*n* = 84) to people who reported currently being attack-free (*n* = 22) in terms of their CH-QoL total scores. People who reported recurring CH attacks showed significantly higher CH-QoL scores compared with people currently having no CH attacks (Table 4). Similarly, people with chronic CH showed significantly worse CH-related QoL compared with people with episodic CH (Table 4).

**Table 4:** Group comparisons CH-QoL (total score)

**Disease activity**	**No attacks** ***M (SD)***	**Recurring attacks** ***M (SD)***	***t***-**test**	**Cohen’s ** ***d***
CH-QoL (total score)	42.3 (35.3)	64.8 (20.0)	*t*(24.6) = − 2.88, *p* = .008	0.94
**Subtype of CH**	**Episodic CH** ***M (SD)***	**Chronic CH** ***M (SD)***	***t-*** **test**	**Cohen’s ** ***d***
CH-QoL (total score)	54.9 (28.0)	65.0 (22.0)	*t*(95.0) = − 2.05, *p* = .043	0.40

*Note* Welch *t*-tests were conducted because Levene test suggested a violation of the assumption of equal variances

Finally, we found a statistically significant medium-sized correlation between the CH-QoL total score and the number of CH attacks in the past month (*r* = 0.31, *p* = .001). This implies that a higher frequency of CH attacks is associated with decreased CH-specific QoL.

### Interpretation for clinical use

Each item requires a response on a 5-point scale (0 = never, 1 = occasionally, 2 = sometimes, 3 = often, 4 = always). Since each item addresses a facet of *reduced* QoL (e.g., item 1: Avoided leaving the house), higher values reflect a lower QoL (referring to the last month). For a total score (“CH-QoL total sum”), the values of all items have to be added. The score of subscale 1 (“restriction of activities of daily living”) is built by adding the values of item 1–9, 19, 20, and 22–27. The score of subscale 2 (“impact on mood and interpersonal relationships”) is built by adding the values of item 10–18, 21, and 28. A comparison of both subscales is provided by adjusting for the different number of items (score of subscale 1/17 and score of subscale 2/11). A cut-off from which, for example, an indication for targeted treatment due to significantly reduced QoL can be derived, does not yet exist. However, to assess the meaning of a patient’s individual values, it is possible to compare them with the means and standard deviations in our sample (Supplementary Table S1).

## Discussion

The assessment of health-related QoL provides a holistic view of the state of health that goes beyond purely biomedical aspects, and it is important especially in people with chronic diseases. The CH-QoL allows a specific assessment of QoL in people with CH. The original English version has demonstrated good reliability and validity, comprising four subscales (labeled “restriction of activities of daily living”, “impact on mood and interpersonal relationships”, “pain and anxiety”, and “lack of vitality”). This study aimed to develop a German-language version and to determine the psychometric properties of this version.

Our results confirm the validity and reliability of the German-language version of the CH-QoL. However, factor analysis (EFA) yielded two subscales (factors), which is in discrepancy to the study from Abu Bakar and colleagues [13], who identified four subscales (factors). Since the items of the two subscales of our study largely match the content of the first two subscales of Abu Bakar and colleagues, we have adopted each the names (subscale 1: “restriction of activities of daily living”, subscale 2: “impact on mood and interpersonal relationships”). The items 1–9 are assigned to subscale 1 in the same way as for Abu Bakar and colleagues. As a difference, the items 19, 20, 22–27 are also assigned to subscale 1. Since the items 19 (“forgetfulness”) and 20 (“neglect of appearance”) had a sufficiently high loading on subscale 1 in our study and also fit very well with the area “restriction of activities of daily living” in terms of content, we consider an allocation to subscale 1 to be justifiable. The items 22–27 had a sufficiently high loading on subscale 1 as well. Some of these items also fit very well with the area “restriction of activities of daily living” in terms of content, for example, item 26 (“problems in concentrating”) and item 27 (“unable to think clearly”). The items 22 (“unbearable pain”) and 23 (“dread of persistent headache”) form a separate subscale (subscale 3, “pain and anxiety”) in Abu Bakar and colleagues. However, this subscale could not be replicated in our analysis. Questionnaires with several subscales should contain at least three items per subscale [34]. Subscales that consist of only a few items have a low probability of being replicated [34]. Since the subscale “pain and anxiety” consists of only two items, this may be an explanation for the lack of replication. We subsume these two items also under the first scale, even if their content is not perfectly fitting. Regarding subscale 2, we have identified the items 10–18, 21, and 28 as belonging to it. Because of the high overlap (Abu Bakar and colleagues: items 10–18, 21), we label subscale 2 similarly as “impact on mood and interpersonal relationships”. In contrast, item 28 (“tense or anxious”, originally subsumed under the subscale “lack of vitality”) is now subsumed under subscale 2. This assignment fits also in terms of content since being tense or anxious can be perceived as an impairment of mood as well. In summary, our factor analysis resulted in two clearly interpretable subscales, which correspond to the first two subscales of the original English version in terms of their names, but under which a broader spectrum of items is subsumed.

The pattern of correlations with established questionnaires for similar constructs (i.e., general health-related QoL, psychosocial burden, state of health, disability) confirms the convergent validity of the German version of the CH-QoL. All correlations are in the expected direction. Higher values in the CH-Qol reflect a lower disease-specific QoL. The correlations with the SF-36 (where higher values reflect a higher QoL) are negative and in the medium to high range (*r* = –0.36 to –0.80). As well, the correlations with the MSQ (where higher values also reflect a higher QoL) are negative and in the medium to high range (*r* = –0.66 to –0.88). In contrast, Abu Bakar and colleagues report positive correlations of the CH-QoL with the MSQ. This is because MSQ values were not recoded (i.e., inverted) in Abu Bakar and colleagues. As a further difference, the amount of correlation is higher in our study (range *r* = –0.66 to –0.88 vs. *r* = 0.28 to 0.65 in Abu Bakar and colleagues). One explanation could be that the low values (0.28, 0.29, 0.41) come from the correlations of the MSQ with the subscale “pain and anxiety”, which consists of only two items. This subscale could not be confirmed in our analysis and is probably not very reliable. The correlations of CH-QoL with the EQ-5D-3L (where higher scores reflect higher impairment of general health) are in the low to medium range (*r* = 0.10 to 0.48), similar to Abu Bakar and colleagues (range *r* = 0.07 to 0.54). Interestingly, most of the correlations with the subscales of the CHS (which aims to assess psychosocial burden in people with CH) [11] are in the low to medium range (except for the correlations with subscale “disability”), which suggests that the CHS captures other facets compared to the CH-QoL. This supports the recommendation in the guidelines of the International Headache Society for controlled clinical trials in CH, which suggests the use of both instruments [15]. The low, not statistically significant correlations of the CH-QoL with the subscale “Openness” of the NEO-FFI-30 confirm divergent validity.

People who indicated recurring attacks as well as people with chronic CH each reported a significantly lower QoL than people who were attack-free or with episodic CH. In addition, we found a statistically significant medium-sized correlation between the CH-QoL total score and the number of CH attacks in the past month. These findings can also be interpreted as an indication of the validity of the CH-QoL.

One strength of the study is the high quality of the data set. By excluding people with a too fast processing time and an incorrect response to the IRI, implausible data could be removed. By using established questionnaires that assess similar constructs, good statements on validity can be made, which is another strength. Since the participants of our study were recruited in a clinical setting (headache centers, patient support groups) it is not surprising that chronic CH is more present (51.9%) as expected from epidemiological studies. Despite extensive recruitment efforts, the target sample size (*N* = 280) was not achieved. After the response rate had stagnated, it was decided to end the recruitment phase due to limitations on project resources. A sample size of *N* ≥ 280 would have been the optimal prerequisite for conducting an EFA. Thus, the results should be interpreted with caution. A confirmatory factor analysis (which requires an even larger sample) [20], was not carried out, which represents a limitation. A cross-sectional design was chosen for reasons of research economy. Since no repeat measurement was carried out, statements on test-retest reliability are not possible, which represents a further limitation. General disadvantages of online surveys can be mentioned as limitations as well (e.g., skewed attributes of the internet population). Despite the listed limitations, we firmly believe that this questionnaire can already be a valuable resource to clinicians and sheds dearly needed light on patients’ declines in QoL. Since economic approaches are desirable in clinical care, the reduction of the CH-QoL to two subscales should be an improvement. For the future, it would be worthwhile to determine a cut-off for both the total score and the two subscales, from which the indication for a targeted treatment to improve QoL could be derived. For example, if a patient exceeds the cut-off in subscale 2, targeted interventions to improve mood and interpersonal relationships could be applied. Thus, future studies should strive to determine a cut-off, for example by using an external criterion. Moreover, future studies should examine the scale in larger clinical samples to further investigate the factor structure. To determine the test-retest reliability, a design with repeated measurement would be useful. In addition, the sensitivity to change should be determined, which requires an intervention between the measurements.

## Conclusions

The German version of the CH-QoL has very good psychometric properties (high reliability and validity), and it is therefore suitable for the assessment of disease-specific QoL in people with CH in German-speaking countries. The two-factor structure (subscale 1: “restriction of activities of daily living”, subscale 2: “impact on mood and interpersonal relationships”) is sufficient to depict the QoL of people with CH. Both the CH-QoL and the CHS should be used as PROMs to assess the burden in people with CH, as they cover different aspects.

## Electronic supplementary material

Below is the link to the electronic supplementary material.

Supplementary Material 1

Supplementary Material 2

Supplementary Material 3

## Data Availability

De-identified data of the current study is available online at the Open Science Framework (OSF): Klan, T. (2024). German language adaptation of the Cluster Headache Quality of Life Scale (CH-QoL). https://osf.io/hfvpb/?view_only=f818656982fa4d468b3513da249d2f90.

## Abbreviations

CH: Cluster headache
CHIQ: Cluster Headache Impact Questionnaire
CH-QoL: Cluster Headache Quality of Life Scale
CHS: Cluster Headache Scales
EFA: Exploratory factor analysis
EQ-5D-3L: EuroQoL 5 Dimensions Questionnaire
IRI: Instructed response item
MINRES: Minimum residual
MSQ v2.1: Migraine-Specific Quality of Life Questionnaire Version 2.1
NEO-FFI-30: NEO-Five-Factor-Inventory (30-Item-Short-Version)
PROMs: Patient-reported outcome measures
QoL: Quality of life
RMSEA: Root mean square error of approximation
RSI: Relative speed index
SF-36: Short Form 36 Health Survey Questionnaire
SRMR: Standardized root mean square residual
TLI: Tucker-Lewis Index
VAS: visual analog scale
