# Supplementary material for: German language adaptation of the Cluster Headache Quality of Life Scale (CH-QoL)
Source: BMC Neurol. 2024 Nov 7;24:433. doi: 10.1186/s12883-024-03923-6 (PMC11542247; doi:10.1186/s12883-024-03923-6)
Supplement: Supplementary file 1 — Supplementary Material 1 [file 12883_2024_3923_MOESM1_ESM.docx]

**Fragebogen zur Lebensqualität bei
Clusterkopfschmerz (CH-QoL)**

Wie viele Clusterkopfschmerzattacken hatten Sie während des letzten Monats?*

* eine ungefähre Angabe (ggf. unter Zuhilfenahme eines Kopfschmerztagebuchs) reicht aus

Anzahl: ______

Bitte geben Sie an, wie oft der Clusterkopfschmerz WÄHREND DES LETZTEN MONATS verschiedene Bereiche Ihres Lebens beeinflusst hat, indem Sie jede der folgenden Aussagen bewerten.

Bitte kreuzen Sie für jede Aussage nur ein Kästchen an. Bitte lassen Sie keine Aussage unbeantwortet.

| **Wie oft ist es während des**  **letzten Monats aufgrund von Clusterkopfschmerz**  **vorgekommen, dass Sie:** | **nie** | **selten** | **manchmal** | **oft** | **immer** |
| --- | --- | --- | --- | --- | --- |
| 1. es vermieden haben, das Haus zu verlassen. | □ | □ | □ | □ | □ |
| 1. es aufgrund der Unvorhersehbarkeit von Clusterkopfschmerz vermieden haben, Pläne zu machen (z.B. Urlaub). | □ | □ | □ | □ | □ |
| 1. sich nicht in der Lage dazu gefühlt haben, berufliche Aufgaben zu erfüllen. | □ | □ | □ | □ | □ |
| 1. Schwierigkeiten hatten, an Freizeitaktivitäten teilzunehmen (z.B. Kino- oder Theaterbesuche). | □ | □ | □ | □ | □ |

| **Wie oft ist es während des**  **letzten Monats aufgrund von Clusterkopfschmerz**  **vorgekommen, dass Sie:** | **nie** | **selten** | **manchmal** | **oft** | **immer** |
| --- | --- | --- | --- | --- | --- |
| 1. überfüllte und laute Orte gemieden haben (z.B. Restaurants, öffentliche Verkehrsmittel). | □ | □ | □ | □ | □ |
| 1. das Gefühl hatten, dass die Schwere des Clusterkopfschmerzes Sie bei Ihren täglichen Aktivitäten beeinträchtigt. | □ | □ | □ | □ | □ |
| 1. sich weniger an familiären Aktivitäten beteiligt haben   (z.B. Umgang mit Kindern, Urlaubsplanung). | □ | □ | □ | □ | □ |
| 1. nicht in der Lage dazu waren, soziale Kontakte zu pflegen/   Zeit mit der Familie und Freunden zu verbringen. | □ | □ | □ | □ | □ |
| 1. nicht in der Lage dazu waren, Ihre Tagesziele zu erreichen sowie Ihre Alltagsaufgaben und den Haushalt zu erledigen. | □ | □ | □ | □ | □ |
| 1. sich von anderen weniger respektiert gefühlt haben. | □ | □ | □ | □ | □ |
| 1. Schwierigkeiten in engen Beziehungen hatten. | □ | □ | □ | □ | □ |

| **Wie oft ist es während des**  **letzten Monats aufgrund von Clusterkopfschmerz**  **vorgekommen, dass Sie:** | **nie** | **selten** | **manchmal** | **oft** | **immer** |
| --- | --- | --- | --- | --- | --- |
| 1. das Gefühl hatten, Ihrer Familie oder Freunden zur Last zu fallen. | □ | □ | □ | □ | □ |
| 1. sich nach einer Clusterkopfschmerzattacke wegen Ihres Aussehens  (z.B. Schwellung/Rötung der Augen, Schwitzen im Gesicht) verunsichert und unwohl gefühlt haben. | □ | □ | □ | □ | □ |
| 1. das Gefühl hatten, dass andere Ihre Clusterkopfschmerzen nicht ernst nehmen. | □ | □ | □ | □ | □ |
| 1. sich aggressiv gefühlt haben. | □ | □ | □ | □ | □ |
| 1. negative Gedanken über sich selbst hatten, das Selbstvertrauen verloren haben oder sich wertlos gefühlt haben. | □ | □ | □ | □ | □ |
| 1. den Wunsch hatten, sich etwas anzutun oder sich umzubringen. | □ | □ | □ | □ | □ |
| 1. reizbar, ungeduldig oder weniger nachsichtig waren. | □ | □ | □ | □ | □ |

| **Wie oft ist es während des**  **letzten Monats aufgrund von Clusterkopfschmerz**  **vorgekommen, dass Sie:** | **nie** | **selten** | **manchmal** | **oft** | **immer** |
| --- | --- | --- | --- | --- | --- |
| 1. vergesslich waren  (z.B. Termine verpasst haben). | □ | □ | □ | □ | □ |
| 1. nicht in der Lage dazu waren, sich um Ihr Äußeres zu kümmern  (z.B. zu baden oder zu duschen, sich zu schminken, die Kleidung zu wechseln). | □ | □ | □ | □ | □ |
| 1. sich isoliert, einsam oder verletzlich gefühlt haben. | □ | □ | □ | □ | □ |
| 1. den Schmerz als unerträglich empfunden haben, wenn dieser nicht behandelt wurde. | □ | □ | □ | □ | □ |
| 1. befürchtet haben, dass der Kopfschmerz nicht mehr aufhört. | □ | □ | □ | □ | □ |
| 1. sich energielos und ständig müde gefühlt haben. | □ | □ | □ | □ | □ |
| 1. sich aufgrund nächtlicher Clusterkopfschmerzattacken übermüdet, erschöpft oder weniger konzentrationsfähig gefühlt haben. | □ | □ | □ | □ | □ |

| **Wie oft ist es während des**  **letzten Monats aufgrund von Clusterkopfschmerz**  **vorgekommen, dass Sie:** | **nie** | **selten** | **manchmal** | **oft** | **immer** |
| --- | --- | --- | --- | --- | --- |
| 1. Schwierigkeiten hatten, sich zu konzentrieren (z.B. beim Zeitung lesen, Fernsehen). | □ | □ | □ | □ | □ |
| 1. nicht klar denken konnten. | □ | □ | □ | □ | □ |
| 1. sich angespannt oder ängstlich gefühlt haben. | □ | □ | □ | □ | □ |
| Bitte bewerten Sie nun Ihre Lebenszufriedenheit INSGESAMT, indem Sie auf der nachfolgenden Skala einen senkrechten Strich an der zutreffenden Stelle setzen:    \| \|  Überhaupt nicht zufrieden Sehr zufrieden | | | | | |

Kuhn, H., Petzke, T., Schreiber, M.-C., Gaul, C., Witthöft, M. & Klan, T. (2024). Deutschsprachige Version der Cluster Headache Quality of life scale (CH-QoL).

Abu Bakar, N., Torkamani, M., Tanprawate, S., Lambru, G., Matharu, M., & Jahanshahi, M. (2016). The development and validation of the Cluster Headache Quality of life scale (CHQ). *The Journal of Headache and Pain*, *17*, 1-9. <https://doi.org/10.1186/s10194-016-0674-1>

Cappon, D., Ryterska, A., Akram, H., Lagrata, S., Cheema, S., Hyam, J., ... & Jahanshahi, M. (2021). The sensitivity to change of the cluster headache quality of life scale assessed before and after deep brain stimulation of the ventral tegmental area. *The Journal of Headache and Pain*, *22*, 1-8. <https://doi.org/10.1186/s10194-021-01251-5>
