# Supplementary material for: German language adaptation of the Cluster Headache Quality of Life Scale (CH-QoL)
Source: BMC Neurol. 2024 Nov 7;24:433. doi: 10.1186/s12883-024-03923-6 (PMC11542247; doi:10.1186/s12883-024-03923-6)
Supplement: Supplementary file 2 — Supplementary Material 2 [file 12883_2024_3923_MOESM2_ESM.docx]

**CLUSTER HEADACHE QUALITY OF LIFE QUESTIONNAIRE (CHQ)**

How many times have you experienced a cluster headache attack during the last month? _______________

Please complete the following items to indicate how often cluster headache has affected various aspects of your life DURING THE LAST MONTH

Please check only one box for each item. Do not leave any item blank.

| **Due to cluster headache, during the last month how often have you:** | **Never** | **Occasionally** | **Sometimes** | **Often** | **Always** |
| --- | --- | --- | --- | --- | --- |
| 1. Avoided leaving the house |  |  |  |  |  |
| 1. Avoided making plans due to unpredictability of cluster headache (e.g. holidays) |  |  |  |  |  |
| 1. Felt unable to complete duties at work |  |  |  |  |  |
| 1. Had difficulty in getting involved in leisure activities (e.g. go to the movies, theatre) |  |  |  |  |  |
| 1. Avoided crowded and noisy places (e.g. restaurants, public transport) |  |  |  |  |  |
| 1. Felt that the severity of cluster headache affected your daily activities |  |  |  |  |  |
| 1. Been less involved in family affairs (e.g. interaction with children, planning vacations) |  |  |  |  |  |
| 1. Been unable to socialize/spend time with friends and family |  |  |  |  |  |
| 1. Been unable to achieve your daily goals and carry out routines and chores |  |  |  |  |  |
| 1. Felt less respected by others |  |  |  |  |  |
| 1. Had problems with close personal relationship |  |  |  |  |  |
| 1. Felt you were a burden on family and friends |  |  |  |  |  |
| 1. Felt self-conscious and uncomfortable about your appearance after a cluster headache attack (e.g. swelling/redness of eyes and facial sweating) |  |  |  |  |  |
| 1. Felt that others are dismissive of your cluster headaches |  |  |  |  |  |
| 1. Felt aggressive |  |  |  |  |  |
| 1. Felt bad about yourself, lost self-confidence or felt worthless |  |  |  |  |  |

| **Due to cluster headache, during the last month, how often have you:** | **Never** | **Occasionally** | **Sometimes** | **Often** | **Always** |
| --- | --- | --- | --- | --- | --- |
| 1. Felt like harming yourself or suicidal |  |  |  |  |  |
| 1. Been irritable, impatient or less tolerant |  |  |  |  |  |
| 1. Been forgetful (e.g. missed appointments) |  |  |  |  |  |
| 1. Been unable to take care of your appearance (e.g. take a bath or shower, put make-up on, change clothes) |  |  |  |  |  |
| 1. Felt isolated, lonely or vulnerable |  |  |  |  |  |
| 1. Found your pain is unbearable if untreated |  |  |  |  |  |
| 1. Dreaded that the headache would not go away |  |  |  |  |  |
| 1. Felt lacking in energy and constantly tired |  |  |  |  |  |
| 1. Felt sleepy, worn out or less able to concentrate due to nocturnal attacks of cluster headache |  |  |  |  |  |
| 1. Had problems concentrating (e.g. reading newspaper, watching TV) |  |  |  |  |  |
| 1. Been unable to think clearly |  |  |  |  |  |
| 1. Felt tense or anxious |  |  |  |  |  |

Please rate your overall satisfaction with your life by placing a vertical line on the scale below at an appropriate point

___________________________________________

| |

Not at all satisfied Very satisfied

Abu Bakar, N., Torkamani, M., Tanprawate, S., Lambru, G., Matharu, M., & Jahanshahi, M. (2016). The development and validation of the Cluster Headache Quality of life scale (CHQ). *The Journal of Headache and Pain*, *17*, 1-9. <https://doi.org/10.1186/s10194-016-0674-1>

Cappon, D., Ryterska, A., Akram, H., Lagrata, S., Cheema, S., Hyam, J., ... & Jahanshahi, M. (2021). The sensitivity to change of the cluster headache quality of life scale assessed before and after deep brain stimulation of the ventral tegmental area. *The Journal of Headache and Pain*, *22*, 1-8. <https://doi.org/10.1186/s10194-021-01251-5>
