# Supplementary material for: German language adaptation of the Cluster Headache Quality of Life Scale (CH-QoL)
Source: BMC Neurol. 2024 Nov 7;24:433. doi: 10.1186/s12883-024-03923-6 (PMC11542247; doi:10.1186/s12883-024-03923-6)
Supplement: Supplementary file 3 — Supplementary Material 3 [file 12883_2024_3923_MOESM3_ESM.docx]

**Supplementary Table 1. Mean (standard deviation) of the CH-QoL total score and subscales**

|  | Total sample  (*N* = 106)  *M* (*SD*) | Episodic CH  (*n* = 51)  *M* (*SD*) | Chronic CH  (*n* = 55)  *M* (*SD*) |
| --- | --- | --- | --- |
| CH‑QoL total score ^a^ | 60.2 (25.4) | 54.9 (28.0) | 65.0 (22.0) |
| Subscale 1 (restriction of activities of daily living) ^b^ | 40.3 (16.5) | 37.0 (18.6) | 43.4 (13.7) |
| Subscale 2 (impact on mood and interpersonal relationships) ^c^ | 19.8 (10.2) | 18.0 (10.3) | 21.6 (9.8) |

^a^Range of possible values: 0 to 112

^b^Range of possible values: 0 to 68

^c^Range of possible values: 0 to 44
